# Supplementary material for: Based on abnormal fluctuations in user-side flow simulation analysis of low- and medium-pressure gas pipeline leakage monitoring
Source: PLoS One. 2022 Jul 25;17(7):e0270290. doi: 10.1371/journal.pone.0270290 (PMC9312409; doi:10.1371/journal.pone.0270290)
Supplement: Attachment — Submitted filename: PONE-D-22-03731 review comments.docx [file pone.0270290.s002.docx]

This work aims at the monitoring of low- and medium-pressure gas pipeline leakage by analyzing abnormal fluctuations of user-side flow. This paper is attractive to practitioners in the pipeline industry. Although the research results of this paper are generally correct, some conditions and conclusions are not well combined with practice. Therefore, this article needs to be slightly modified.

Major comments:

1. Page 6, line 9-12: The assumptions that the gas is an ideal gas and the flow process is isothermal flow, will lead to a certain error between the simulation results and the actual situation.

2. Page 7, line 20-22: It can be seen from Figure 2 that the assertion that "the time required for this trend to reach stability under various working conditions is 6s, 14s, 6s, 8s, 8s and 6s respectively" is not correct. It is recommended to carefully refer to figure 2 Determine the time required for this trend to reach stability under various working conditions.

3. Page 9, line 3-10: According to figure 2, not all flow fluctuations on the user side caused by leakage conditions return to a stable state within 10 seconds. The duration of flow fluctuations on the user side caused by the first five leakage conditions is 10-20s, and the flow fluctuations on the user side caused by the sixth leakage condition do not tend to be stable within the displayed time interval. The optimal monitoring period shall be determined according to the actual time to reach stability

4. Page 10, line 1-7: (1) It is not explained that the calculation of flow disturbance amplitude in Figure 3 is based on the flow data of which of the six leakage conditions.

(2) From Figure 3, the conclusion that "no matter where the leakage is in the pipeline, the flow disturbance amplitude tends to be consistent within 2 ~ 3 seconds after the leakage" is not accurate. For example, when the leakage occurs at node T, the flow disturbance amplitude is 2% in the second second and tends to 0 in the third second. It is suggested to modify or delete this clause
