# Supplementary material for: Based on abnormal fluctuations in user-side flow simulation analysis of low- and medium-pressure gas pipeline leakage monitoring
Source: PLoS One. 2022 Jul 25;17(7):e0270290. doi: 10.1371/journal.pone.0270290 (PMC9312409; doi:10.1371/journal.pone.0270290)
Supplement: Attachment — Submitted filename: Review comments.docx [file pone.0270290.s004.docx]

Thank author for your serious reply to my comments. These changes you have made can make this article meet the publishing requirements. However, if you can make a precise explanation of which working condition the data in Figure 3 comes from, it will make the article more rigorous.
