# Supplementary material for: Based on abnormal fluctuations in user-side flow simulation analysis of low- and medium-pressure gas pipeline leakage monitoring
Source: PLoS One. 2022 Jul 25;17(7):e0270290. doi: 10.1371/journal.pone.0270290 (PMC9312409; doi:10.1371/journal.pone.0270290)
Supplement: Attachment — Submitted filename: Response to Reviewers.docx [file pone.0270290.s005.docx]

# Reviewer #1: Review comments:

1. The last part of the paper (conclusion and prospect) are too complicated. It is suggested to summarize and highlight the innovation.

Re: Thanks to the reviewer for the suggestion about simplifying the conclusion and highlighting the innovation. We’ve revised the conclusion and prospect carefully as follows.

# “Conclusion

(1) It is found in this paper that when the middle and low-pressure gas pipeline leaks, the user-side flow changes from fluctuating to stable and shows a certain change cycle and law, which is related to the location of the leak. Based on this, we propose a method to determine the location of the leak. In addition, this method can detect and locate leakage using abnormal fluctuations on the user-side.

(2) Besides the location of the pipeline leak, there are many other influencing factors, such as air supply pressure and flow, and the size of the leak. It is found that the higher the air supply pressure, the larger the air supply flow, the smaller the leakage aperture, and the longer the duration of flow fluctuations, but they can all recover from fluctuations to a stable state within 10s. Therefore, the best monitoring period for user flow is set to 10s.

(3) The location of the leak can be roughly determined based on the abnormal flow fluctuations within 10s after the leak occurs. When the leakage is in the first 40% of the relative distance from the gas source, the abnormal flow fluctuation first increases and then decreases, otherwise, the flow decreases monotonously.

(4) The location of the leak can be carefully judged based on the magnitude of abnormal flow fluctuation in the flow rate for 10 consecutive seconds after the leak occurred, due to the closer the leakage point is to the user side, the more significant the abnormal flow fluctuation will be.

# Discussion

This research can provide a basis for the detection and location of the leakage of medium and low-pressure gas pipelines. It can be expected that the method proposed can be embedded in the intelligent analysis system of the medium and low-pressure gas pipeline network, changing the leakage detection method of manual inspection. The detection pattern of medium and low-pressure gas leakage accidents will be changed from passive notification to active monitoring.

The simulation method proposed in this paper still has the following deficiencies：

(1) There are some mechanical errors in user-sidepressure and flow measurement that affect the leak detection accuracy of the theoretical formula directly. Domestic regulations allow measuring equipment to have a measurement error of 1% to 3% [23], but the structural defects of the instrument will further enlarge the measurement error as the service life of measuring equipment increases.

(2) Restrictions on pipeline laying conditions. Most of the outdoor gas pipelines in our country are laid underground. These pipelines must consider the soil pore leakage rate [24]. The theoretical formula adopted in this paper is simplified, which defaults to the natural gas pipelines asoverhead pipelines. And the relevant soil pore leakage rate is set to 1 [25], this indicator could be rectified in further research.

(3) It can be considered to calculate multiple parameters (pressure difference, flow difference, and temperature difference) under the same working condition simultaneously and realize the method of identifying leakage working conditions based on multivariate data fusion.

(4) The assumption that the gas is an ideal gas and the flow process is isothermal flow will lead to a certain error between the simulation results and the actual situation. The influence of gas type and temperature on the change of user flow rate should be considered in further study to make the simulation results closer to reality.

”

# Reviewer #2: Review comments:

2. Thank author for your serious reply to my comments. These changes you have made can make this article meet the publishing requirements. However, if you can make a precise explanation of which working condition the data in Figure 3 comes from, it will make the article more rigorous.

Re: Thanks to the reviewer for this suggestion to improve our work, our description of the case in Figure 3 was not clear before and is explained below. Figure 3 shows the flow disturbance amplitude of user-end caused by different leakage locations within 8S after the leakage occurs under condition 1. Condition 1 is the basic condition of the simulation experiments in this paper, and all leakage parameters are the basic settings (see Table 2). It can be seen in Figure 3 that the flow fluctuation of node T returns to 0 first, that is, the instantaneous flow reaches a balance, and the fluctuation range of node B is the last to return to 0. This rule can be verified from Figure 2. It can be seen from Figure 2 that under any working condition, the changes of user-end flow at different leak locations show similar laws: when the leak location is at the T-node close to the user-end, the flow first reaches a balanced state. The farther the leak location is from the user-end, the longer it takes to reach equilibrium. When the leak occurs at node B, it takes the longest time for the flow to reach the equilibrium state.
